# A training paradigm to enhance performance and safe use of an innovative neuroendovascular device

**DOI:** 10.3402/jmahp.v4.33248

**Published:** 2016-11-09

**Authors:** Donald R. Ricci, Thomas R. Marotta, Howard A. Riina, Martina Wan, Joost De Vries

**Affiliations:** 1 Division of Cardiology, University of British Columbia, Vancouver, BC, Canada; 2 Evasc Medical Systems Corporation, Vancouver, BC, Canada; 3 Diagnostic and Therapeutic Neuroradiology, St Michael’s Hospital, University of Toronto, Toronto, Ontario, Canada; 4 Department of Neurosurgery, NYU School of Medicine, NYU Langone Medical Center, New York, NY, USA; 5 Department of Neurosurgery, Radboud University Nijmegen, Nijmegen, The Netherlands

**Keywords:** endovascular, medical devices, training, education, intracranial aneurysm, stroke, proctoring

## Abstract

Training has been important to facilitate the safe use of new devices designed to repair vascular structures. This paper outlines the generic elements of a training program for vascular devices and uses as an example the actual training requirements for a novel device developed for the treatment of bifurcation intracranial aneurysms. Critical elements of the program include awareness of the clinical problem, technical features of device, case selection, and use of a simulator. Formal proctoring, evaluation of the training, and recording the clinical outcomes complement these elements. Interventional physicians should embrace the merits of a training module to improve the user experience, and vendors, physicians, and patients alike should be aligned in the goal of device training to improve its success rate and minimize complications of the procedure.

Since the onset of endovascular intervention in the late 1970s, training has been important for the safe and effective use of new devices that were developed to facilitate the return of diseased (congenitally or acquired) or damaged vascular structures to a more normal physiologic state. Adequacy of this training frequently is the difference between success and failure and, more importantly, freedom from complication in endovascular endeavors. As newer endovascular devices become more complex, sophisticated, and innovative, training in their appropriate application and use is even more important. Large government regulatory and nongovernment bodies such as the U.S. Food and Drug Administration (FDA) and the World Health Organization (WHO) have recognized the dilemma of the mismatch between the profusion of medical devices and capacity of the end user to assimilate the information necessary for the proper use of new devices. In particular, training in the nuances of the use of catheter-based technologies becomes even more important as endovascular medicine becomes predominant over surgical intervention in some fields (1, 2).

Although catheter-based diagnostic procedures have existed since the 1930s, with the pioneering experiments of Forssmann and Cournand (3), coaxial catheter-wire techniques were not developed until the 1980s to enable interventional procedures (4). These techniques allowed safe and effective navigation of a microcatheter passed through a guiding catheter and over a guide wire to a site of vascular disease, with the aim of a vascular intervention, in this first instance, balloon angioplasty. And it was not long before triaxial catheter systems (a tube within a tube within a tube, the smallest, in turn, being advanced over a wire) were used to reach deeper and more selectively into the vascular system. Although the fastest growth and greatest application of these techniques initially occurred in the coronary vascular field to deliver devices such as balloons and stents to improve outcomes, these techniques are now extensively used throughout the vascular tree, not only to deliver devices to facilitate repair of blood vessels, per se, but also for correction of congenital and acquired cardiac defects, valvular disorders, for delivery of chemotherapeutic agents, nanoparticles, glues, and thrombolytic drugs (5–11).

## Objective

This paper outlines the generic elements of a training program for vascular devices and uses as an example the actual training requirements for a novel device developed for the treatment of intracranial aneurysms occurring at bifurcations, the eCLIPs device (Evasc Medical Systems Corp., Vancouver, BC, Canada) (12).

## Methods

### Elements of training

#### Awareness of the clinical problem

Training on the use of a device begins with awareness of the clinical problem for which the device is developed to manage. Bifurcation intracranial aneurysms are a major source of morbidity and mortality. Sixty-four percent of all cerebral aneurysms occur at arterial bifurcations (13). Worldwide prevalence of unruptured intracranial aneurysm is 3.2% in the general population, but this number may be higher in females, older patients, and patients with an affected family history or certain genetic conditions (14). A systematic review of all reports on prevalence of intracranial aneurysms concluded that rupture occurs in 1–2% of all intracranial aneurysms per year and results in a subarachnoid hemorrhage (15). This hemorrhage may be life-threatening, with a mortality rate of 25–50%, or it may cause significant cognitive, physical, and/or psychological disabilities in nearly 50% of the survivors, entailing a considerable impact on their quality of life (16). The eCLIPs device was developed to address the most complex subset of this disease.

#### Technical features of device: alignment with the anatomy of the clinical problem

**Fig. 1:**
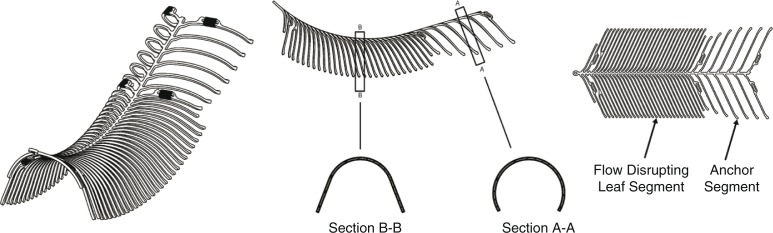
eCLIPs device: three-dimensional view (left), side view (middle), plan view (right).

The next step is to confirm that the design of the device and the expected benefits of the design elements will address the clinical problem. The functional attributes of the eCLIPs device depend on a spine-rib design (Fig. 1) that cannot shorten upon deployment; an anchor segment that provides stability of the device *in situ*; and a dense leaf segment that bridges the neck of the aneurysm to allow for coil retention, flow disruption away from the aneurysm sac, and endothelial growth. The device can be safely retracted back into the microcatheter and repositioned or removed before detachment. All these features combine to produce healing of the aneurysm to return the bifurcation to its original physiologic state: the device is fully incorporated into the vessel wall, leaving no metal in either the main vessel or side branches, exteriorizes the aneurysm from the circulation, and does not impede access to side branches. Hence, the eCLIPs device allows for physiologic remodeling of the bifurcation aneurysm (12).

Reviewing the device’s indications for use (IFU), as determined by various regulatory bodies (e.g., CE Mark), is a requirement before choosing the device. Important in the analysis of the use of a new device is the comparison of its design elements against those of current technologies developed for similar indications. By this process, the interventional physician will have a better understanding of the pros and cons of using various technologies in specific situations.

Evasc’s training program presents each eCLIPs product to the physician with a discussion of the product’s description, the specific function accruing to each of the design features, and its IFU.

A laminated image set, ‘eCLIPs Introduction Images’, illustrates the eCLIPs treatment concept compared to other technologies.

#### Case selection

Having identified the clinical problem, the need for a solution, and recognition that the proposed device has design features that can provide a functional solution to the problem, the next step is selection of a case to be managed by the device. Although the interventionalist may use the foregoing process to make an informed decision to use the device in a chosen case, communication with mentors and other colleagues, clinical specialists employed by the device’s vendors, and even use of an interactive App (e.g., eCLIPs MD, Apple Store) may be effective methods to refine case selection and avoid inappropriate cases, especially early in the interventionalist’s experience with the device.

#### Use of simulator

**Fig. 2:**
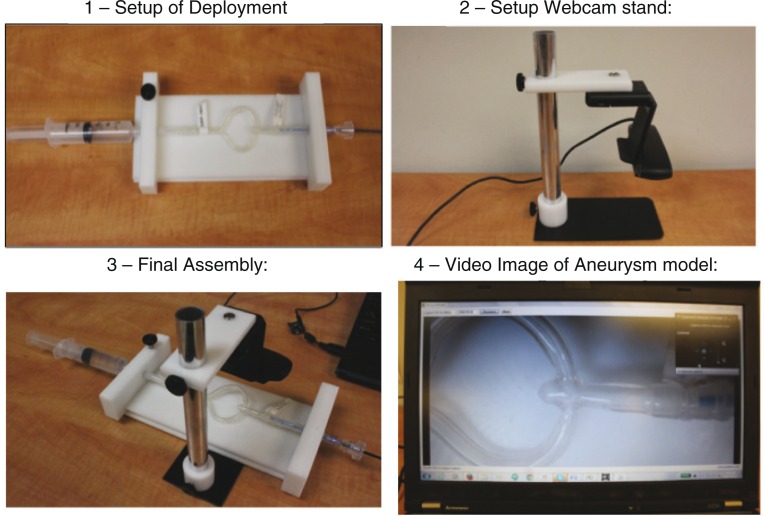
Illustrations of benchtop setup of aneurysm model simulator.

Hands-on experience with a system model that simulates clinical anatomy, with options for a variety of anatomic models that illustrate a spectrum of anatomic variations should be a prerequisite to clinical use in the laboratory. A well-constructed model (Fig. 2), using live video rather than radiographic imaging, can usually supplant the need for trialing the device in an animal model that is expensive, wasteful, and contrary to environmental and animal protection mores. A simulation model is compact, mobile, can be erected in minutes, and uses custom-made aneurysm models that can be constructed to mimic the precise anatomy of a planned case.

**Fig. 3:**
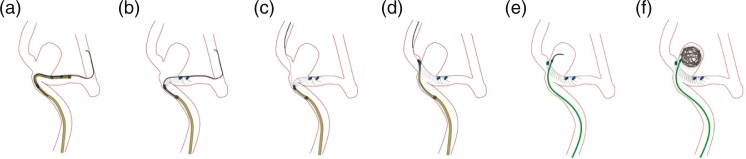
Animated eCLIPS deployment sequence.

In Evasc’s hands-on demonstration, the trainee uses the system model setup to deliver, deploy, position, and then detach an eCLIPs device while following the eCLIPs product’s IFU. This simulated use training is performed under the direction of the trainer, either a physician or a clinical specialist. There is also an animated demonstration of how the eCLIPs device is deployed (Fig. 3) and how the other products (catheter, detacher) work.

During this simulated use training, the trainer refers to the eCLIPs Simulated Use Training Checklist section of the Physician Training Form so that documentation exists at each step.

### Clinical procedural aspects

#### Proctoring

After becoming versed in the use of the device in a simulator, if feasible the next step should be for the trainee to attend a proctor’s case as an observer. The trainee is encouraged to share several possible cases with the proctor for discussion of lesion suitability. This is best achieved at the original session to allow interactive learning, but it can occur any time afterward. Also, if feasible in a short time thereafter the trainee should proceed with a case (having discussed case selection, reviewed the simulator experience, and gained the usual patient consent). The clinical specialist and a proctor should attend the case, the former to provide a refresher to the simulator experience immediately before the case and the latter to provide detailed procedural nuances as the case proceeds.

#### Device-related procedural details

Each device and its delivery and detachment mechanisms will be more or less unique to the device, and even after review in a simulator they should require attention to detail during the procedure.

Evasc’s training for eCLIPs introduces the hypotube of the delivery portion of the eCLIPs device and how to use a mobile wire through the hypotube. During preparation, the focus is on the following (12): exposing the device once situated in a branch vessel (Fig. 3a and b); obtaining second branch access (Fig. 3c); confirmation of correct orientation of device, retracting device into the sheath as necessary, and review orientating and positioning (Fig. 3d); and detachment and subsequent coiling, as necessary (Fig. 3e and f).

### Evaluation of training

Evaluating the training by choosing prespecified outcomes that satisfy the training goals is important in the whole process of device training. These may be categorized as follows:

1. Proctor adjudication of case selection, procedural strategy, adherence to procedural strategy, and compliance with IFU.
2. Outcome of device deployment elements, including successful guide wire placement and use as a rail in the triaxial system; success of delivery catheter (microcatheter) placement; precision of device placement (axial and rotational alignment); and adjuvant coiling if necessary.
3. Procedural outcome is evaluated by a combination of successful deployment, successful coiling, angiographic evaluation of outcome, and absence of complications.
4. Finally, it is important to record data and critical elements of the procedure and outcome for quality review and comparison with the use of other technologies.
  Evasc Medical has designed its physician training program for the eCLIPs products to meet the following objectives:
  - Understand the purpose of each eCLIPs product
  - Obtain experience with the eCLIPs product through a simulated use training model
  - Obtain experience with the eCLIPs product through proctored clinical cases

A physician is considered trained on the eCLIPs product once the first two objectives have been met and the first few clinical cases are performed with a proctor. It is recommended that the first five clinical cases using the eCLIPs product be proctored by a physician designated by Evasc; however, some physicians may require more or fewer cases with a proctor depending on the experience, inherent skill and personality of the physician, time elapsed between cases, and other factors. Ultimately, the requirement for a proctor being present or not is determined by Evasc in consultation with the trainee physician and a proctor.

A Training Form documenting all of the above-mentioned requirements is considered a record of the physician’s completion of training on the eCLIPs product.

### Clinical follow-up

Clinical follow-up is the ultimate method to review not only the training program but also the efficacy and safety of the device. Follow-up must include patient status, other clinical comorbidities, angiographic outcome, and presence or absence of complications.

Evasc’s requirements for follow-up of eCLIPs implantation include the following:

1. Patient neurologic status
2. Other procedure-related clinical morbidity/mortality (e.g., bleeding)
3. Angiographic outcome
  - Raymond Score (17)
  - Evidence for device migration
  - Evidence for vessel trauma
4. All data are collected in either a prospective registry or a prospective clinical trial

## Results

Since 2013, with the first eCLIPs implantation under Canada’s Special Access Program through CE Mark approval in 2014, and through to July 31, 2016, Evasc’s training program for the eCLIPs device has initiated training for 23 neuro-interventionalists at 17 clinical sites. This training has yielded 36 clinical implants at 13 sites with no device-related complications (18). The clinical efficacy of the eCLIPs device seems, in this early experience, to be favorable. Three proctors have participated in the training program, in aggregate having implanted at least 20 devices. Two clinical specialists have been trained.

Because the primary proctor (TRM) is based in Toronto, Canada, travel has made it impossible to attend cases in person in Europe in four instances. In these cases (none the first attempted by the trainee), proctoring was done via video Skype, with a clinical specialist in the procedure room coordinating the communication between the proctor and the trainee.

The decision to grant sign off on the Training Record, indicating that the trainee has successfully completed the training, has been made for six neurointerventionalists.

Feedback from the trainees, their clinical laboratory staff, proctors, and clinical specialists has generally been favorable and has resulted in a continuous quality improvement of the training manual and procedures.

## Discussion

Creation and implementation of training modules for the use of medical devices, their applications, and the outcomes of training are not discrete requirements in any regulatory approval process, such as the Medical Device Directorate or the FDA. For example, the Council Directive of the European Economic Community (EEC) 93/42/EEC (19) contains the rule that ‘each device must be accompanied by the information needed to use it safely and properly, taking account of the training and knowledge of the potential users, and to identify the manufacturer’. But the information is restricted to ‘the details on the label and the data in the instructions for use’. Thus, the onus is on the vendor or manufacturer to develop fulsome training guidelines to be sure that the interventionalist can apply the IFU within a step-by-step technical framework.

Such formal and specific guidelines are typically effected through the Risk Mitigation principles in a Quality Systems doctrine (20). EN ISO 14971:2012 provides a process for managing risks associated with medical devices. A serious risk, of course, results from technical misuse of the device, either because of failure to follow the IFU or lack of knowledge of any nuances to the step-by-step nature of its use, as distinguished from similar use devices.

Many interventionalists, because of their high level of skill, large experience with the use of triaxial catheter techniques, and/or extensive history with the use of multiple new medical devices over a long period, become inured to reading details of the IFU or perhaps, more importantly, to assuming that use of a new device is ‘intuitive’. An excellent example of the cavalier attitude to the detail of the IFU is the widespread use of devices ‘off label’, meaning they are being applied to solve a problem for which the device was not explicitly designed or approved. The FDA guidance on this matter suggests that ‘if physicians use a product for an indication not in the approved labeling, they have the responsibility to be well informed about the product …’ (21) and further asserts that ‘use of a marketed product in this manner when the intent is the “practice of medicine”’ is appropriate, contingent on local ethics reviews or other oversight. In the neurointerventional sphere, the use of dual ‘Y- or T-stenting’ for management of bifurcation aneurysms is probably the most notable use of a device off-label to achieve a solution to a complex, otherwise untreatable condition (22).

Interventional physicians should embrace the merits of a training module to improve the user experience, and vendors, the physicians, and the patients alike should be aligned in the goal of device training to improve its success rate and minimize complications of the procedure. WHO has concluded that endovascular devices are ideally suited for ‘virtual reality’ simulation training such as that described herein over other methods such as use of animal models (23). Our data cannot provide information on the direct effect of device training upon improved success or mitigation of complication without an assessment of these effects in a cohort where no training has occurred. The ethics of such a trial would indeed be questionable. To suggest that training in the use of a new interventional device is of value may, indeed, be intuitive.

## Conclusions

A formal device training program, containing multiple elements, is a necessity for successful application of the device to a clinical therapeutic procedure. Elements of a generic program have been presented, exemplified by the specific program for an innovative device developed for the management of bifurcation intracranial aneurysms.
